# To: Behavior of lung ultrasound findings during spontaneous breathing trial

**DOI:** 10.5935/0103-507X.20180020

**Published:** 2018

**Authors:** Jacobo Bacariza Blanco, Antonio M. Esquinas

**Affiliations:** Unidade de Terapia Intensiva, Hospital Garcia De Orta EPE - Almada, Portugal.; Unidade de Terapia Intensiva, Hospital Meseguer - Murcia, Espanha.

**To the Editor**

The new century brought a "new" kid on the block to the field of intensive care. Ultrasound (US) came from the radiology departments, and it has renewed intensive care medicine. Focused on the critical care patient and with the intensivist as a performer, US has set a new paradigm for medicine. Due to its increasing relevance, in 2009,^(1)^ the American College of Chest Physicians and La Société de Réanimation de Langue Française published a statement on competence in order to set the minimum standards to achieve the appropriate learning skills of the four main US approaches: thoracic, cardiac, vascular and abdominal. Since then, ultrasound is no longer an option but mandatory for the new intensivists. However, in addition to its promising potential, much is yet unknown.

With great attention, we read the remarkable paper by Antonio et al.^(2)^ on how this consideration enlarges the knowledge spectrum by focusing on lung ultrasound (LUS) application on a spontaneous breathing trial (SBT) during weaning from mechanical ventilation. Fifty-seven enrolled patients received LUS before and after the SBT. In conclusion, the authors observed a loss of lung aeration during SBT in patients who failed the weaning.

Nevertheless, two things need to be considered.

Firstly, the B-lines^(3)^ are not always pathological while seen below three per field between two rib spaces at the Merlin space, especially when located at the PLAPS point (lower lung scanning). Rockets, which are greater than 2 B-lines, are always related to a lung parenchyma mismatch due to the interstitial space syndrome. The rockets are observed in association with or without lung sliding and define two different ultrasound patterns, B (present slide) and B´ (absent slide). Both of them are related to interstitial syndrome, but B is transudate and thus cardiogenic, while B´ is exudative and thus related to pneumonia. These are two clinical conditions with specific clinical and treatment approaches and, of course, different considerations during weaning from mechanical ventilation. 

Secondly, the rockets have a demonstrated sensitivity of 97% and specificity of 95% in detecting acute pulmonary edema, and furthermore, as described in the aforementioned paper, the B-pattern is an Extra Lung Water Index (ELWI) validated measurement.^(3,4)^ In short, this LUS pattern is a reliable tool to identify cardiogenic pulmonary edema. However, different B-patterns have already been identified, based on the number of rockets per field. These include septal rockets (3 - 4), ground glass (6 - 8) and the Birolleau variant (contiguous B-lines with no anechoic space in between). Each one translates as pulmonary edema, but with an increasing degree of severity and, again, different conditions with specific features to bear in mind when treated or weaned. In order not to undermine US test simplicity, the authors suggest not to document some additional cardiac, vein, or pleural effusion US features. We do not agree because those features could have serious implications on the patient outcome, specifically the diastolic cardiac dysfunction assessment. When the clinician is trained, he/she can perform the measurement accurately without consuming much time.

Finally, we do not agree with the authors' position on four-region scans. There is no LUS gold standard at the present. However, the Blue Protocol accuracy^(5)^ is not only validated in acute respiratory failure but also has notable sensitivity and specificity values. The blue protocol is based on six-region scans.^(6)^

Further clinical trials and research are in demand to understand the full potential of lung ultrasound.

*Jacobo Bacariza Blanco* *Intensive Care Unit, Hospital Garcia De Orta EPE - Almada,
Portugal.* *Antonio M. Esquinas* *Intensive Care Unit, Hospital Meseguer - Murcia,
Spain.*
